# Enzyme-Antibody-Modified Gold Nanoparticle Probes for the Ultrasensitive Detection of Nucleocapsid Protein in SFTSV

**DOI:** 10.3390/ijerph17124427

**Published:** 2020-06-19

**Authors:** Yuqin Duan, Wei Wu, Qiuzi Zhao, Sihua Liu, Hongyun Liu, Mengqian Huang, Tao Wang, Mifang Liang, Zhiyun Wang

**Affiliations:** 1 School of Life Sciences, Tianjin University, Tianjin 300072, China; dyqaitd@163.com (Y.D.); 17865515170@163.com (Q.Z.); lsh158158@tju.edu.cn (S.L.); hy19950518@yeah.net (H.L.); hmq961012@163.com (M.H.); wangtaobio@tju.edu.cn (T.W.); 2 National Institute for Viral Disease Control and Prevention, Chinese Center for Disease Control and Prevention, Beijing 100000, China; wuwei@ivdc.chinacdc.cn; 3 School of Environmental Science and Engineering, Tianjin University, Tianjin 300072, China

**Keywords:** SFTSV, nucleocapsid protein, gold nanoparticles, sandwich enzyme-linked immunosorbent assay

## Abstract

As humans and climate change continue to alter the landscape, novel disease risk scenarios have emerged. Sever fever with thrombocytopenia syndrome (SFTS), an emerging tick-borne infectious disease first discovered in rural areas of central China in 2009, is caused by a novel bunyavirus (SFTSV). The potential for SFTS to spread to other countries in combination with its high fatality rate, possible human-to-human transmission, and extensive prevalence among residents and domesticated animals in endemic regions make the disease a severe threat to public health. Because of the lack of preventive vaccines or useful antiviral drugs, diagnosis of SFTS is the key to prevention and control of the SFTSV infection. The development of serological detection methods will greatly improve our understanding of SFTSV ecology and host tropism. We describe a highly sensitive protein detection method based on gold nanoparticles (AuNPs) and enzyme-linked immunosorbent assay (ELISA)—AuNP-based ELISA. The optical sensitivity enhancement of this method is due to the high loading efficiency of AuNPs to McAb. This enhances the concentration of the HRP enzyme in each immune sandwich structure. The detection limit of this method to the nucleocapsid protein (NP) of SFTSV was 0.9 pg mL^−1^ with good specificity and reproducibility. The sensitivity of AuNP-based ELISA was higher than that of traditional ELISA and was comparable to real-time quantitative polymerase chain reaction (qRT-PCR). The probes are stable for 120 days at 4 °C. This can be applied to diagnosis and hopefully can be developed into a commercial ELISA kit. The ultrasensitive detection of SFTSV will increase our understanding of the distribution and spread of SFTSV, thus helping to monitor the changes in tick-borne pathogen SFTSV risk in the environment.

## 1. Introduction

Between 2007 and 2010, a severe febrile illness was associated with gastrointestinal symptoms, thrombocytopenia, leukocytopenia, and high mortality in the eastern and central regions of China [1,2]. This disease is called severe fever with thrombocytopenia syndrome (SFTS) and was caused by the newly discovered bunyavirus (SFTSV) [3]. Subsequently, SFTS was confirmed in South Korea, Japan, and Vietnam [4,5]. Ticks are the vectors for transmission of the virus to humans [6,7].

SFTSV is a negative-chain segmental RNA virus consisting of three fragments (L, M, and S). The L, M, and S segments encode RNA-dependent RNA polymerase, precursors of glycoproteins (Gn and Gc), nucleocapsid (N) proteins, and nonstructural (NS) proteins, respectively [8].

The nucleocapsid protein (NP) is closely related to viral replication [9,10] and is highly immunogenic and conserved. Therefore, NP is often selected as a target for antigen and antibody detection [11].

Several methods of genomic amplification for SFTS diagnosis have been reported, including qRT-PCR, reverse transcription-loop-mediated isothermal amplification assay (RT-LAMP), and reverse transcription-cross-priming amplification coupled with vertical flow visualization [12,13]. However, genome amplification techniques are limited by their need for expensive equipment and technical expertise. The enzyme-linked immunosorbent assay (ELISA) is the most common immunoassay for clinical biomarker detection because of its good specificity, low cost, and simple reading method. Methods for the detection of viral antigens by Ag-capture sandwich ELISA have been described previously, and the sensitivity of this assay is comparable to RT-PCR [14]. The limit of detection for ELISA is 0.1 ng mL^−1^ to 1 µg mL^−1^ [15], and the sensitivity of traditional ELISA cannot screen for ultra-low concentrations of biomarkers in the early stages of certain diseases. Therefore, there is an urgent need to develop ultrasensitive detection methods for the different types of biomarkers.

In this context, nanotechnology offers many ways to improve detection sensitivity. Nanomaterials, such as gold nanoparticles (AuNPs) [16], magnetic beads [17], graphene oxide [18], Polyamidoamine dendrimer (PAMAM) [19], silica [20], and plasmonic nanoparticles [21] can be used for detection applications [15]. AuNPs are distinguished from other nanoparticles and quantum dots containing harmful heavy metal ions because of their simpler synthesis process and effective surface modification [22]. Their high surface area can carry many biomolecules (such as antibodies, enzymes, or DNA) to produce significant signal enhancement [23,24]. The most common enzyme that can be coupled to AuNPs is horseradish peroxidase (HRP). This has been widely used for detection purposes because of its small size and high stability of chemical modification [22].

AuNPs have been widely used in clinical diagnosis [15,25]. Ambrosi [23] directly conjugated AuNPs with HRP-labeled anti-human IgG antibody and detected 50 pg mL^−1^ of human IgG—this value is 50 times more sensitive than traditional ELISA. Jia [26] and Wu [27] developed a dual-modified gold nanoprobes for enhanced immunoassay using the same experimental principle. In their experiments, they used AuNPs as a bridge between the detection antibody and HRP. The methods they created were one to three orders of magnitude higher than the classical method. The results described in these prior studies all prove that AuNPs have a large capacity for carrying proteins—this is a great advantage in the field of improving detection sensitivity.

A key step in obtaining the AuNP probes is the conjugation of biomolecules to AuNPs. Several parameters, such as surface modification, pH, stabilizers, and addition processes, strongly influence the final coverage and efficiency of biomolecules [28,29]. Studies by Ciaurriz [22] and Aubin-Tam [30] have shown that direct adsorption is the most sensitive and easiest way to prepare probes (the combination of AuNPs and antibodies occurs through noncovalent bonding, and no chemical steps are required). It is more likely to retain the structure of biomolecules than covalently link the proteins. In a study of Ni in 2013 [31], HRP retained only 30% of the catalytic activity after immobilization on the surface of 25 nm AuNPs.

In this study, we used an enzyme-labeled antibody to label the AuNPs. Thus, the enzyme freely moved in solution, which allowing the substrate to be close to the enzyme without encountering steric hindrance from the AuNPs.

## 2. Experimental Section

### 2.1. Materials

We purchased skim milk, bovine serum albumin (BSA), PEG 20000, tween-20, and Tetramethylbenzidine single-component substrate solution (TMB) from Beijing Solarbio Science & Technology Co., Ltd. (Beijing, China). HRP was purchased from Shanghai Yiji Industrial Co., Ltd. (Shanghai, China). Gold (III) chloride trihydrate (HAuCl_4_·3H_2_O) was purchased from Sigma-Aldrich (St. Louis, MO, United States). Sodium periodate, sodium borohydride, and trisodium citrate were obtained from Shanghai Aladdin Industrial Corporation (Shanghai, China). All chemicals were of analytical reagent grade and obtained from the commercial source unless mentioned otherwise. Our laboratory prepared SFTSV recombinant N protein, mouse monoclonal antibody (McAb) (1A1, 1D8, 1H10, 4H1, 3A9), and rabbit polyclonal antibody to NP [32,33]. The NPs were aliquoted and stored at −80 °C until use. HRP-labeled goat anti-mouse secondary antibody was purchased from Tianjin Sanjian Biotechnology Co., Ltd. (Tianjin, China). A QIAamp Viral RNA Mini Kit was purchased from Qiagen (Frankfurt, Germany). TransScript First-Strand cDNA Synthesis SuperMix and TransStart Probe qPCR SuperMix were purchased from Beijing TransGen Biotech Co., Ltd. (Beijing, China). Healthy human serum samples were provided by Weifang Medical University. Microdialysis bags were from Beijing Solarbio Science & Technology Co., Ltd. (Beijing, China). The 96-well polystyrene plates were obtained from Corning Costar (NYC, USA).

We used the following buffers: (1) wash buffer: 0.01% Phosphate buffer solution (PBS) (pH 7.4); (2) storage buffer: PBS with 0.5% BSA, 2.5% sucrose, 50% glycerin, and 0.1% PEG 20000; (3) blocking buffer: PBS with 5% skim milk; and (4) probes dilution buffer: PBS with 5% BSA.

### 2.2. Instruments

We used a transmission electron microscopy (TEOL, JEM-1010, Tokyo, Japan), nanoparticle size and zeta potential analyzer (Malvern Instrument Co., Ltd., Zetasizer nano ZS90, Malvern, United Kingdom), multifunction microplate reader (PerkinElmer, EnSpire Multilabel Reader, Waltham, MA, USA), microplate reader (Tecan, Infinite F50, Switzerland), ultra-small rotary incubator (Haimen Qilin Bell Instrument Manufacturing Co., Ltd., QB328, Haimen City, Jiangsu, China), micro-refrigerated centrifuge (Beckman Coulter, microfuge 22R, CA, USA), and real-time fluorescent quantitative PCR instrument (Roche, LightCycler 96, Basel, Switzerland).

### 2.3. Preparation of Enzyme-Labeled Antibodies

We prepared the enzyme-labeled antibody as described [34]. We dissolved 0.5 mg of HRP in 200 μL of distilled water and added 20 μL of fresh 0.1 M NaIO_4_ solution to it. The mixture was stirred at room temperature in the dark for 20 min. We dialyzed the solution in sodium acetate buffer (pH 4.4) at 4 °C overnight and added 5 μL of 0.2 M carbonate buffer (pH 9.5) to raise the pH of the above aldehyde-formed HRP to 9.0–9.5. Then, we immediately injected 1 mg of monoclonal antibody (replaced the antibody buffer with 200 μL of carbonate buffer in advance) and gently stirred the mixture for 2 h in the dark. We added 20 μL of fresh NaBH_4_ solution (4 mg/mL) and stirred at 4 °C for 2 h. We dialyzed this solution in 0.01 M PBS (pH 7.4) at 4 °C overnight and added an equal volume of saturated ammonium sulfate solution dropwise, which was mixed up and down. After 1 h at 4 °C, the mixture was centrifuged at 3000 rpm for half an hour. We washed the precipitate twice with half-saturated ammonium sulfate solution, and finally resuspended the precipitate with 200 μL 0.01 M PBS (pH 7.4). This solution was dialyzed against 0.01 M PBS (pH 7.4) at 4 °C to remove ammonium ions, and then the solution was centrifuged at 10,000 rpm for 30 min. The supernatant sucked out was the enzyme conjugate.

The antibody had a maximum absorption peak at 280 nm, and HRP contained a hemin IX as a prosthetic group with a maximum absorption peak at 403 nm [35]. We measured the optical density (OD) at 280 nm and 403 nm with a Nanodrop. A high-quality equivalent of pure glycerin (50%) was added and stored at −20 °C. The freezing point of 50% glycerol was lower than −20 °C, which protected the activity of the antibody by avoiding the repeated freezing and thawing of the antibody [36].

The molar ratio of HRP to antibody (E/P) was calculated as follows [34]:

HRP (mg/mL) = OD403 nm × 0.4;(1)

IgG (mg/mL) = (OD280 nm − OD403 nm × 0.3) × 0.62;(2)

E/P = (HRP × 4)/IgG.(3)

### 2.4. Indirect ELISA

We performed an indirect ELISA to determine the titer of the five McAb raised against NP. First, we coated 100 μL of antigen (10 μg mL^−1^) into each well and incubated them overnight at 4 °C. We then washed the wells six times with PBS. The wells were blocked with 200 μL of skim milk. After washing, we added 100 μL of each dilution (1:1000, 1:4000, 1:16,000, 1:64,000, 1:256,000, 1:1,024,000) of five monoclonal antibodies to each well. We then washed the wells six times with PBS. Next, we added 100 μL of the prepared HRP-conjugated goat anti-mouse antibody and incubated the samples at 37 °C for 60 min. After washing, we added 100 μL TMB solution to promote the reaction. Finally, we measured the optical density using an ELISA reader after stopping the reaction with 100 μL 1 M HCl.

### 2.5. Synthesis and Characterization of AuNPs

Many studies have examined detection using antibody-modified AuNPs [22,37,38,39]: these AuNPs usually have a plasmon absorption peak at 520 nm and a particle size of about 20 nm. The smaller AuNP is too small to effectively conjugate enough antibody, whereas the larger AuNP is easy to precipitate and difficult to wash off from the 96-well plate [40]. Therefore, we chose an AuNP particle size of 20 nm based on previous experience. We prepared the 20 nm AuNPs using the Turkevich method [41]. All glass was first immersed in aqua regia for 1 h, washed three times with deionized water, and then dried at 150 °C. Next, 50 mL of 0.01% HAuCl_4_ solution was boiled, and 3.5 mL of 1% trisodium citrate solution was quickly added to the boiling solution under rapid stirring (500 rpm/min). The citrate ion acted as a reducing agent for the formation and stabilization of AuNPs, preventing its aggregation. The solution was left to stir and cool when the solution turned dark red indicating the formation of AuNPs. The quality of the particles was monitored by ultraviolet-visible (UV−vis) spectrophotometer, zeta potential analysis, and transmission electron microscope.

### 2.6. Gold Aggregation Experiment

As mentioned previously [23], we adjusted the pH of 1 mL of the AuNPs solution to 8.0 with a K_2_CO_3_ (0.1 M) solution and dispensed the AuNPs into a 96-well plate (200 μL). We added different amounts of antibody to the AuNP solution at a final concentration of 10, 15, 20, 25, 30, 35, and 40 μg mL^−1^. This was then mixed and allowed to stand for 10 min. Next, 30 μL of 10% NaCl solution was added and allowed to stand for 2 h. We measured the absorption spectrum of the AuNPs solution in each well using a microplate reader. We then placed the 96-well plate on a piece of white paper and observed the color change. As above, we adjusted the pH of the AuNPs solution to 7.0, 7.5, 8.0, 8.5, and 9.0 with 0.1 M K_2_CO_3_ and 0.1 M HCl. We then added the antibody to a final concentration of 12 μg mL^−1^. The pH at which the color of the AuNPs remained red was the optimum pH.

### 2.7. Preparation of HRP-McAb-AuNPs Probes

We adjusted the pH value of the AuNP solution to 7.0–8.0. We added the McAb-HRP to the previously noted AuNP solution to the final concentration of 12 μg mL^−1^. After that, the mixture was agitated slowly overnight at 4 °C. PEG 20,000 was added to the resulting solution to a final concentration of 0.1% and incubated for 30 min in room temperature. The solution was centrifuged for 40 min with a centrifugal force of 10,000 g to remove the unbound antibodies, HRP and PEG. The solution was stored at −20 °C in a storing buffer for further use. We monitored the quality of the particles with UV−vis spectrophotometer, zeta potential analyzer, and transmission electron microscopy.

We performed quantification of HRP immobilized on AuNP probes. To make a standard curve of HRP, we incubated a series of HRP solutions (2 μL) at concentrations of 500, 250, 125, 62.5, 31.25, 15.6, and 7.8 ng mL^−1^ in 500 μL TMB solution for 3 min. Then we measured the absorbance value at 450 nm after stopping the reaction with 500 μL 1 M HCl solution. We determined the amount of HRP on the AuNP probes based on the standard curve of HRP. We calculated the concentration of the AuNP probes by the following formula: c = A450/ε450 (c represents the concentration of the AuNP probes, A450 represents the absorbance of AuNPs at 450 nm, and ε450 is the extinction coefficient of AuNPs [42]). We calculated the number of HRP molecules on each AuNP by dividing the number of HRP molecules by the number of AuNP.

### 2.8. AuNP-Based ELISA

We added 100 μL of rabbit polyclonal antibody against NP with a concentration of 40 μg mL^−1^ to the wells of the plate. The plate was washed six times with PBS after being coated at 4 °C overnight. We then added 200 μL of 5% skim milk to each well and blocked for 2 h at 37 °C. After washing six times with PBS, we added 100 μL of different concentrations of NP to each well and incubated at 37 °C for 1 h. Chicken IgG served as a negative antigen, and the plate was washed six times and then each well was given 100 μL of 20-fold diluted AuNP probes and incubated at 37 °C for 1 h. After washing six times, the wells were given 100 μL TMB solution for 13 min and protected from light at room temperature. We then measured the absorbance at 450 nm after stopping the reaction with 100 μL 1 M HCl.

### 2.9. Detection of Authentic SFTSV by AuNP-Based ELISA

SFTSV at a known titer of 10^6^ TCID_50_/mL were lysed with 1% Triton-X100 for 1 h to obtain NP. The resulting virus lysate was then serially diluted and detected by AuNP-based ELISA. We used the culture supernatant of Vero cells as a negative control and performed the same treatment as we had for the virus sample.

### 2.10. Detection of Authentic SFTSV by qRT-PCR

We used the SFTSV culture supernatant for RNA extraction by the QIAamp Viral RNA Mini kit. We used TransScript First-Strand cDNA Synthesis SuperMix and TransStart Probe qPCR SuperMix for cDNA Synthesis and qRT-PCR, respectively. We performed the qRT-PCR method using qRT-PCR primers and probe as previously described [13]. The cDNA was serially diluted to 1:10^1^, 1:10^2^, 1:10^3^, 1:10^4^, 1:10^5^, 1:10^6^, 1:10^7^, and 1:10^8^ before being detected with qRT-PCR. We analyzed results using the 2^−ΔΔCt^ method [43,44].

## 3. Results and Discussion

### 3.1. Preparation and Identification of Enzyme-Labeled Antibodies

**Figure 1:**
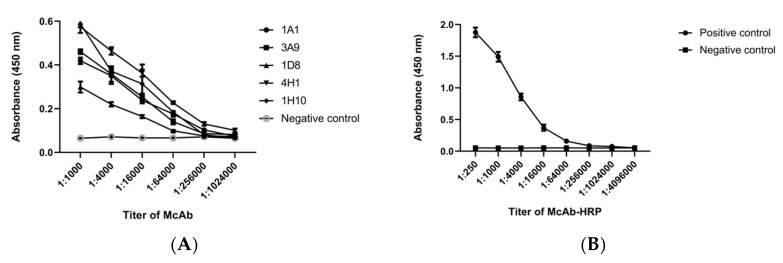
(**A**) The five monoclonal antibodies (1A1, 3A9, 1H10, 4H1, 1D8) selected for titer detection, and (**B**) the results of titration assays of monoclonal antibody horseradish peroxidase (McAb-HRP). Error bars represent the standard deviations (SDs) of two independent assays.

In the indirect ELISA test, the antibody in the sample was sandwiched between the antigen coated on the 96-well plate and the enzyme-labeled secondary antibody. The higher the antibody content was in the sample, the stronger the corresponding absorbance value. The indirect ELISA method was suitable for determining the antibody levels in the samples. We used an indirect ELISA to titer five McAbs. Figure 1A shows that the titer of 1A1, 3A9, 1H10, and 4H1 was 1:64,000, and the titer of 1D8 was 1:16,000, according to the principle of P/N ≥ 2.0 [45]. Antibodies with a higher dilution had a higher titer, which meant they had a strong ability to bind antigen. We finally selected 1H10 for HRP labeling.

We labeled 1H10 with HRP using sodium periodate oxidation. The OD values of the McAb-HRP at 280 nm and 403 nm were 0.120 and 0.0787, respectively. As discussed in Section 2.3, the molar ratio of HRP to IgG was calculated to be 2.11. When the molar ratio was between 1 and 2, it was suitable for further use as reported [34], which proved that HRP could be successfully coupled to antibodies. We determined the titer of McAb-HRP [46]. The assay result was still positive till at a dilution of McAb-HRP of 1:64,000, which showed that this labeling method was gentle and McAb-HRP had good enzyme and antibody activity.

### 3.2. Preparation and Characterization of AuNPs Probes

Before we combined the antibody with the AuNPs, we had to determine the correct ratio between the two because this related to the stability of the colloid. When pH > pI, the antibody was negatively charged and repelled the AuNPs. Sufficient antibody could stabilize the AuNPs from 1% NaCl and it would not flocculate, so the plasmon absorption peak of AuNPs could be stabilized at 520 nm, and the absorbance value was also stable. Therefore, as the concentration of McAb-HRP increased, the AuNPs became increasingly stable, and the absorbance value at 520 nm gradually increased and also tended to be stable.

**Figure 2:**
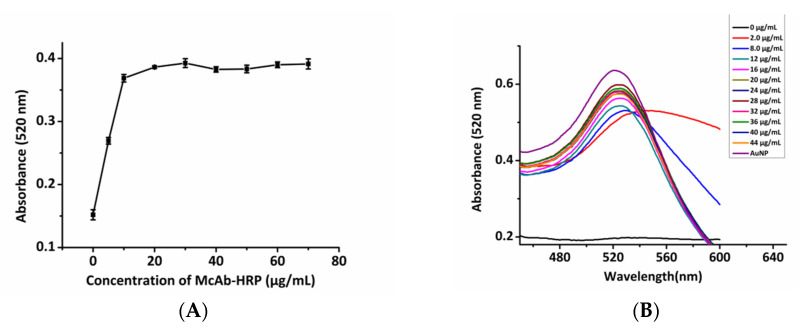

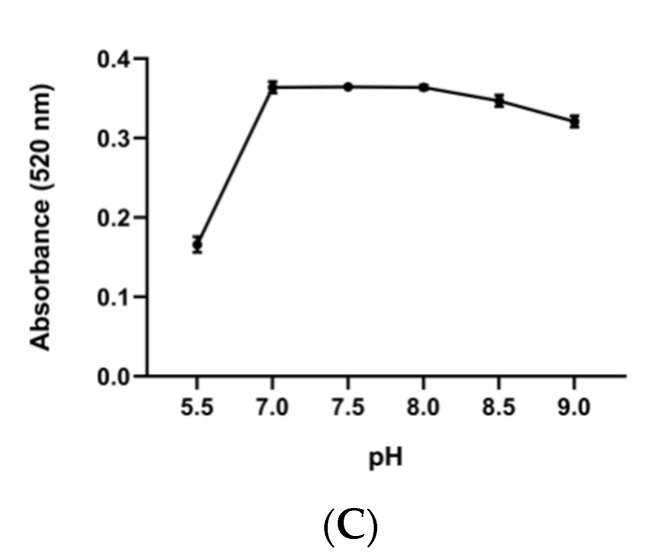
Optimal concentration of McAb-HRP and optimal pH of gold nanoparticle (AuNP) for the conjugation of antibody to AuNPs in gold aggregation experiments. Changes in (**A**) absorbance values and (**B**) AuNP spectra with increasing concentrations of McAb-HRP; and (**C**) absorbance value changes of AuNPs at 520 nm with pH. The error bars show the mean ± SD of two determinations.

The minimum requirement for antibody concentration was the concentration at which gold colloids could remain stable even with the addition of 1% NaCl. This is by far the best choice for the highest efficiency-to-cost ratio in mass production [47]. When the concentration of McAb-HRP was 10 μg mL^−1^, the absorbance value of the AuNPs at 520 nm remained stable (Figure 2A) with no red shift (Figure 2B), suggesting that 12 μg mL^−1^ antibody could stabilize the AuNPs in a high salt environment without aggregation. Higher protein concentrations, however, may have caused crowding and thus affected protein folding and activity [30], which may have caused spatial problems, such as blocking substrate binding or AuNP aggregation [40]. Thus, the optimum antibody concentration for labeled AuNPs was 12 μg mL^−1^. Figure 2C shows the optimized pH at an antibody concentration of 12 μg mL^−1^. The colloidal gold had the highest absorbance value from pH 7.0–8.0, which was a suitable pH range.

**Figure 3:**
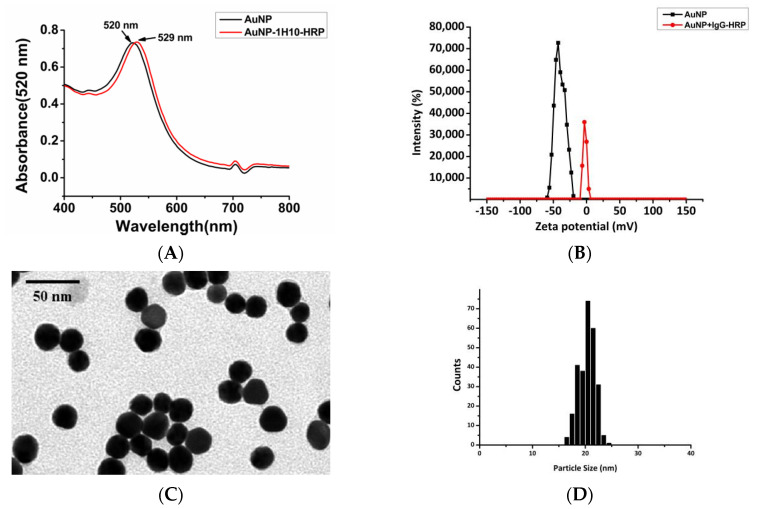

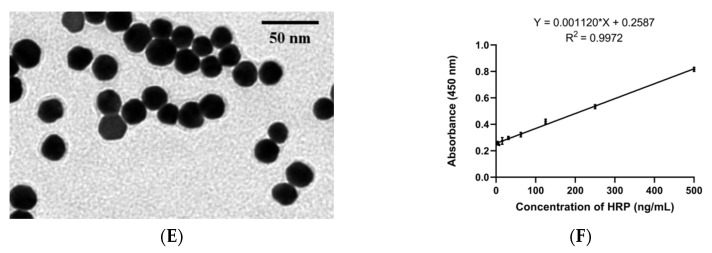
(**A**) Absorption spectrum, (**B**) zeta potential distribution map, (**C**) TEM images of AuNPs, and (**E**) AuNP probe. The 50 nm scale bar applies to all TEM images. (**D**) Statistical histograms of particle size distribution and particle size through the corresponding (**C**) TEM images to obtain the results as a rough approximation of the mean diameter ± the standard deviation. We measured 270 particles were measured for statistics. (**F**) Standard curves of HRP at concentrations of 500, 250, 125, 62.5, 31.25, 15.6, and 7.8 ng mL^−1^ diluted in PBS buffer. The data represent the average ±SD from at least two independent assays.

We synthesized AuNPs with a particle size of 20 nm. We determined the dispersibility and particle size of the AuNP probes by Transmission electron microscope (TEM) and Absorption spectrum. Figure 3A shows that the plasmon absorption peak of the AuNPs was red shifted from 520 nm before labeling the antibody to 529 nm after labeling. This spectral shift was caused by a local refractive index change caused by the protein adhesion layer, which was consistent with previous reports [48]. Figure 3B shows that the zeta potential of the AuNPs was negative (−39.6 mV), and this occurred because the surface of the AuNPs was negatively charged. The absolute value of the AuNPs became smaller after the antibody was attached (−1.96 mV). The TEM data (Figure 3C) showed that the particles were spatially separated, and the average diameter of the AuNPs in the sample was almost the same. We counted 270 nanoparticles and made histograms of particle size distribution and particle size through the corresponding TEM images. The results showed that the average particle size of the gold nanoparticles was 20.4 nm ± 1.6 nm (as shown in Figure 3D). The TEM data (Figure 3E) showed a circle of material outside the AuNPs after the antibody was attached, which was consistent with the phenomenon observed by Woods [49]. The conjugates were homogeneous with high reproducibility (see the adsorption peak in Figure 3A and narrow size distribution in Figure 3E). These data demonstrated that the antibody was successfully coupled to AuNPs.

The antibody loading density was an essential parameter for quantitative measurements. The HRP standard curve in Figure 3F indicated that the concentration of HRP was 3.93 μg mL^−1^ (89.32 nM) on the probes. The amount of IgG was approximately equal to 42.33 nM, and the molar concentration of AuNPs was 2.15 nM. Therefore, there were approximately 19 IgG and 41 HRP per AuNPs (i.e., AuNPs:IgG:HRP = 1:19:41), which proved that AuNPs could conjugate multiple enzyme-labeled antibodies, leading to signal amplification.

### 3.3. AuNP-Based ELISA

**Figure 4:**
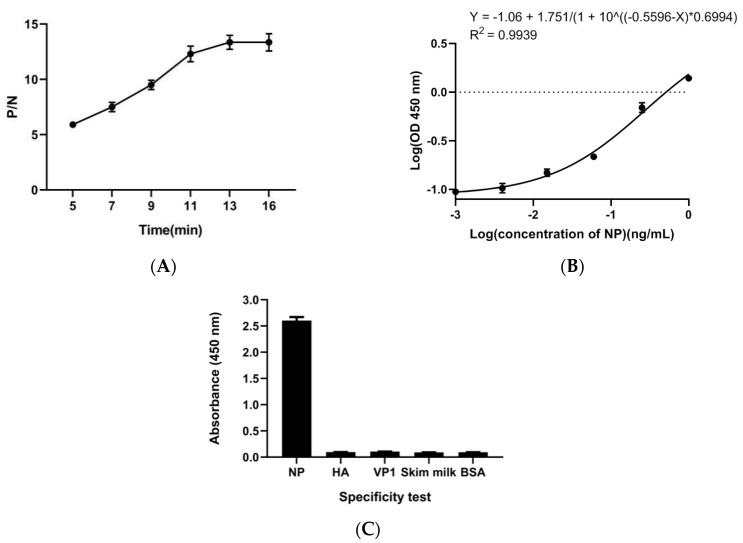
AuNP-based enzyme-linked immunosorbent assay (ELISA). (**A**) Optimization of color development time under optimized conditions in Table 1, and (**B**) calibration curve for the absorbance versus concentration of NP from 0.00025 ng mL^−1^ to 1.0 ng mL^−1^. The absorbance intensity as the ordinate and the concentration of NP as the abscissa. (**C**) Specificity test. The specificity test was completed with NP (3.9 ng mL^−1^), BSA, skim milk, viral protein 1 of Enterovirus D68 (VP1), and Hemagglutinin (HA) (200 ng mL^−1^). All samples were prepared in duplicate.

We set the concentration of the coated antibody in the AuNP-based ELISA to 10, 20, and 40 μg mL^−1^, the antigen concentration was 1 ng mL^−1^, and the probes were diluted to 1:2.5, 1:5, 1:10, and 1:20. When the dilution of McAb-HRP was 1:20 (Table 1) and the concentration of the coated antibody was 40 μg mL^−1^, the P/N value peaked and stabilized. We optimized the coloration time of the ELISA, and the results showed that the P/N value plateaued at 13 min (Figure 4A). Under these conditions, the 4 PL model equation based on AuNP-based ELISA was Y = −1.06 + 1.751/(1 + 10^((−0.5596 − X) * 0.6994)) with a coefficient correlation *R*^2^ = 0.9939 (Figure 4B). The detection limit of the method was 0.9 pg mL^−1^ based on LOD = X + 3SD, which was lower than the previously reported detection limits of traditional ELISA (100 pg mL^−1^) [1]. This proved that AuNPs could carry more HRP molecules leading to greater signal amplification. Ambrosi [24] directly conjugated AuNP with HRP-labeled anti-human IgG antibody. His results showed that even coupling ten HRPs on AuNPs could increase the sensitivity by 50 times. Li [39] coupled five HRPs to AuNPs to increase sensitivity by 20-fold versus traditional ELISA. We coupled 40 HRPs to AuNPs, and the sensitivity increased by 100 times. We also controlled the OD value of the negative control below 0.1, which was one of the reasons for the increased sensitivity.

Two unrelated proteins (VP1 and HA) purified from *Escherichia coli*, skim milk, and BSA were selected for specific detection. The concentration of these unrelated proteins was 200 ng mL^−1^, and the concentration of NP was 3.9 ng mL^−1^. Figure 4C shows that although the concentration of the unrelated protein was much larger than the concentration of NP, only the NP had a strong signal, indicating that our method was highly specific for the detection of NP protein [27].

### 3.4. Detection of NP in Human Serum

We diluted the healthy human serum 100 times, which in turn reduced the sample amount required for analysis [50]. We added three different amounts of NP antigen to the diluted serum to give the different final concentrations; the recovery values are shown in Table 2. The recovery rate was in the range of 80% to 120% [51] demonstrating that detection of NP in human serum was feasible. The complex human serum with a mixture of proteins and other interfering substances did not influence the NP detection. Therefore, the proposed probe could be used for NP-mediated detection in clinical samples.

### 3.5. Detection of Authentic SFTSV via a AuNP-Based ELISA and qRT-PCR

**Figure 5:**
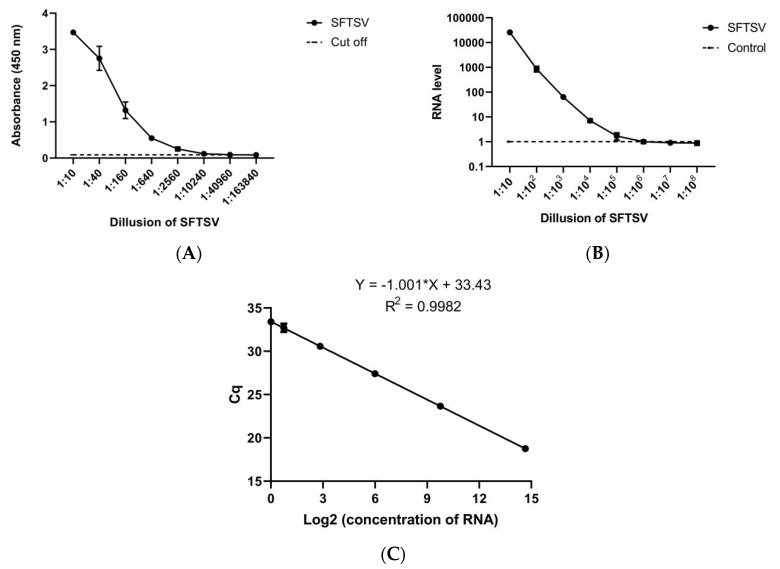
(**A**) Detection of authentic SFTSV via AuNP-based ELISA; qRT-PCR. (**B**) The RNA level corresponding to each dilution. The abscissa is the dilution of cDNA, and the ordinate is the RNA level for each dilution. (**C**) Calibration curve for the quantification cycle [53] (Cq) versus RNA level.

qRT-PCR is the most sensitive technique for gene detection and quantitation currently available. We detected NP gene by qRT-PCR to validate presence and the titer of SFTSV in samples. After proving that the method had a good recovery rate, we compared the sensitivity of the AuNP-based ELISA with the sensitivity of qRT-PCR. In Figure 5A, the highest dilution of the lysate that could be detected was 1:163,840 according to LOD = X + 3SD. Figure 5B,C show that the highest dilution of the lysate that could be detected by qRT-PCR was 1:100,000, according to LOD = LOB + 1.645σ_low concentration sample_ [52]. Therefore, this new method was comparable to traditional qRT-PCR for detecting SFTSV. Meanwhile, the main reagents and probes used in qRT-PCR were very expensive, and the main materials in the AuNPs-based ELISA can be inexpensively prepared in the laboratory, so this method had obvious Economic advantages. We also tried to detect the virus with commercial ELISA kits, but it failed, which indirectly showed that our method was better than the commercially available kits.

### 3.6. Repeatability and Stability Test

**Figure 6:**
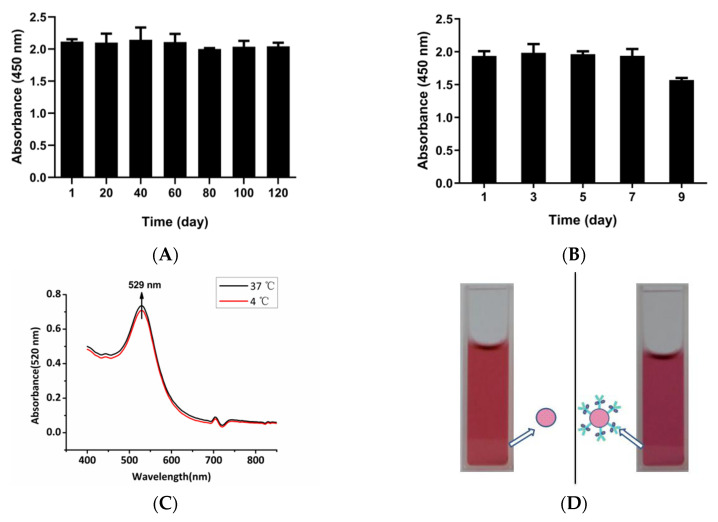
Absorbance changes of the system for the detection of NP after the probe is placed at 4 °C (**A**) and 37 °C (**B**) for different days; (**C**) Absorption spectrum of AuNPs probes stored at 37 °C for seven days and 4 °C for four months. (**D**) Appearance of the AuNPs versus AuNPs probe. The dashed lines indicate the cut-off values (mean + 3 × SD) for each experiment.

We detected different NP concentrations through the same and different batches of microplates. The results are shown in Table 3. The coefficient of variation of each sample was less than 8%, which indicated that the established sandwich ELISA method had good intragroup and good intergroup repeatability [54]. We evaluated the stability of the probes as described [39,55]. The antigen (3.9 ng mL^−1^) was detected by the probes, and the corresponding absorbance value remined stable after about four months at 4 °C (Figure 6A) and for seven days at 37 °C (Figure 6B). The OD value of the unrelated protein remained below 0.1. After nine days of storage at 37 °C, the detection effect was reduced to 81% of the original. We measured the absorption spectra of AuNPs probes stored at 37 °C for seven days and 4 °C for four months. If particle size increases, the wavelength of surface plasmon resonance related absorption will shifts to longer, redder wavelengths. The plasmon absorption peaks of the two probes were still at 529 nm (Figure 6C). It showed that the AuNPs probe had no obvious aggregation phenomenon. We did not observe an aggregation of AuNPs probes with the naked eye under these two temperature conditions. The solution remained translucent and red (Figure 6D), which indicated that the probes were stable during storage.

## 4. Conclusions

AuNP-based ELISA is a highly sensitive protein detection method. In this study, we modified AuNPs with an HRP-labeled monoclonal antibody. We used AuNPs as both a carrier and an amplifier. The detection limit of this method for the NP of SFTSV was 0.9 pg mL^−1^, which was as sensitive as traditional ELISA, and the procedure for detection was as simple as traditional ELISA. The method established here also was comparable to traditional qRT-PCR method for detecting authentic SFTSV. The method provided a good standardization technique and proved to be simple and reliable. No further sample preparation steps were required after the supernatant was collected. Furthermore, this method could detect other antigens by altering the detecting antibody. The techniques described herein exhibited many desirable advantages, including sensitivity, accuracy, and no need for complex equipment. Therefore, this technology demonstrated good potential for reliable early diagnoses of diseases and will contribute to the prevention and control of tick-borne diseases.

## Figures and Tables

**Table 1:** The checkboard titration to determine the concentration of capture antibody and AuNP probes. Comparison of P/N values of the capture antibody with different concentrations and AuNP probes with different dilutions.

Concentration of Coated Antibody (μg/mL)	Dilution of AuNPs Probes			
	1:20	1:10	1:5	1:2.5
10	1.63	1.49	1.49	4.90
20	12.40	12.73	8.15	7.54
40	13.41 ^a^	10.75	8.50	1.13

Note: The table shows the positive per negative (P/N) ratio; ^a^ Represents the suitable P/N ratio from this study.

**Table 2:** Recoveries of NP from spiked human serum samples.

Samples ^a^	Spiked Concentration (ng/mL)	Detected Concentration ^b^	Recovery (%)
1	0.06	0.05	83.30
2	0.25	0.29	116.00
3	1.0	0.88	88.00

*Note:*^a^ Samples 1, 2, and 3 were human serum samples spiked with three different concentrations of NP: 0.06 ng mL^−1^, 0.25 ng mL^−1^, 1.0 ng mL^−1^, respectively. ^b^ Each sample was analyzed two times, and the results are the average values.

**Table 3:** Measurement of intra- and intergroup variations of the AuNP-based ELISA.

Variation	Concetration of NP (ng/mL)	n	CV%
Intra-group variation	1.0	8	6.62
	0.25	8	5.24
	0.06	8	7.12
Inter-group variation	1.0	8	3.49
	0.25	8	4.46
	0.06	8	3.77

*Note*: *n*: number of replicates; CV: coefficient of variation.
